# Disrupted salience network functional connectivity and white-matter microstructure in persons at risk for psychosis: findings from the LYRIKS study

**DOI:** 10.1017/S0033291716001410

**Published:** 2016-07-11

**Authors:** C. Wang, F. Ji, Z. Hong, J. S. Poh, R. Krishnan, J. Lee, G. Rekhi, R. S. E. Keefe, R. A. Adcock, S. J. Wood, A. Fornito, O. Pasternak, M. W. L. Chee, J. Zhou

**Affiliations:** 1 Center for Cognitive Neuroscience, Neuroscience and Behavioral Disorder Program, Duke-NUS Medical School, National University of Singapore, Singapore; 2 Research Division, Institute of Mental Health, Singapore; 3 Office of Clinical Sciences, Duke-NUS Medical School, Singapore; 4 Department of Psychiatry and Behavioral Sciences, Duke University, Durham, NC, USA; 5 Center for Cognitive Neuroscience, Duke University, Durham, NC, USA; 6 School of Psychology, University of Birmingham, Edgbaston, UK; 7 Department of Psychiatry, Melbourne Neuropsychiatry Centre, University of Melbourne and Melbourne Health, Victoria, Australia; 8 Monash Clinical and Imaging Neuroscience, School of Psychology and Psychiatry & Monash Biomedical Imaging, Monash University, Australia; 9 Departments of Psychiatry and Radiology, Brigham and Women's Hospital, Harvard Medical School, Boston, MA, USA; 10 Clinical Imaging Research Centre, the Agency for Science, Technology and Research and National University of Singapore, Singapore

**Keywords:** At risk for psychosis, functional connectivity, LYRIKS, salience network, transition to psychosis, white-matter microstructure

## Abstract

**Background:**

Salience network (SN) dysconnectivity has been hypothesized to contribute to schizophrenia. Nevertheless, little is known about the functional and structural dysconnectivity of SN in subjects at risk for psychosis. We hypothesized that SN functional and structural connectivity would be disrupted in subjects with At-Risk Mental State (ARMS) and would be associated with symptom severity and disease progression.

**Method:**

We examined 87 ARMS and 37 healthy participants using both resting-state functional magnetic resonance imaging and diffusion tensor imaging. Group differences in SN functional and structural connectivity were examined using a seed-based approach and tract-based spatial statistics. Subject-level functional connectivity measures and diffusion indices of disrupted regions were correlated with CAARMS scores and compared between ARMS with and without transition to psychosis.

**Results:**

ARMS subjects exhibited reduced functional connectivity between the left ventral anterior insula and other SN regions. Reduced fractional anisotropy (FA) and axial diffusivity were also found along white-matter tracts in close proximity to regions of disrupted functional connectivity, including frontal-striatal-thalamic circuits and the cingulum. FA measures extracted from these disrupted white-matter regions correlated with individual symptom severity in the ARMS group. Furthermore, functional connectivity between the bilateral insula and FA at the forceps minor were further reduced in subjects who transitioned to psychosis after 2 years.

**Conclusions:**

Our findings support the insular dysconnectivity of the proximal SN hypothesis in the early stages of psychosis. Further developed, the combined structural and functional SN assays may inform the prognosis of persons at-risk for psychosis.

## Introduction

Schizophrenia is increasingly viewed as a disease that involves the dysconnectivity of brain networks (Pettersson-Yeo *et al.* 2011; Fornito *et al.* 2012). Functional network architecture characterized by intrinsic connectivity networks (ICNs) has been widely studied in patients with schizophrenia. ICNs are defined as coherent patterns of low-frequency fluctuations in blood oxygen level-dependent signals derived from task-free functional magnetic resonance imaging (fMRI; Zhou *et al.* 2010). The salience network (SN) is one of the major ICNs and consists of the fronto-insula, anterior cingulate cortex (ACC), orbital frontal cortex (OFC), striatum, as well as limbic structures (Seeley *et al.* 2007). The functional integration of nodes within the SN is crucial to sustain human emotion and cognition, especially during the detection and processing of salient information (Seeley *et al.* 2007; Craig, 2009; Menon & Uddin, 2010). Dysfunction of the SN has been hypothesized to occur in schizophrenia, resulting in inappropriate neural responses to internal and external stimuli. These inappropriate neural responses are thought to eventually lead to positive psychotic symptoms such as hallucination and delusions (Williamson, 2007; Palaniyappan & Liddle, 2012; Palaniyappan *et al.* 2013; Zhou & Seeley, 2014).

SN-related abnormalities in schizophrenia have been consistently found in neuroimaging studies. A number of imaging studies have reported reductions in gray-matter volume (Saze *et al.* 2007; Fornito *et al.* 2009; Takahashi *et al.* 2009) and task-related activation (Minzenberg *et al.* 2009; Wilmsmeier *et al.* 2010) and connectivity (Schmidt *et al.* 2016) within the insula and ACC in schizophrenia. Task-free fMRI studies have demonstrated reduced functional connectivity (FC) within the insula (Liang *et al.* 2006; Zhou *et al.* 2007), ACC (Boksman *et al.* 2005; Honey *et al.* 2005) and other key SN regions. Diffusion tensor imaging (DTI) has been used to examine altered white-matter (WM) microstructures in schizophrenia. Reduction in DTI indices purported to quantify WM diffusion properties, such as fractional anisotropy (FA) and axial diffusivity (AD) in frontal and temporal regions (forming part of the SN) has been reported in schizophrenia (Buchsbaum *et al.* 2006) using voxel-based morphometry (Le Bihan, 2003) and region-of-interest (ROI; Price *et al.* 2005; Li *et al.* 2014) approaches as well as tract-based spatial statistics (TBSS; Lee *et al.* 2012; Liu *et al.* 2013). Furthermore, diffusion tractography studies have directly demonstrated altered structural connectivity within key SN regions in schizophrenia patients (Oh *et al.* 2009; Bracht *et al.* 2014).

Recently, there has been growing interest in the study of adolescents and young adults who are in the putative prodromal stage of schizophrenia and are experiencing subthreshold psychotic symptoms. These individuals are at high risk of transitioning to clinical psychosis over a 36-month period and are labeled as having an ‘At-Risk Mental State’ (ARMS; Yung *et al.* 1996; Fusar-Poli *et al.* 2012). Several functional and structural imaging studies have attempted to examine changes in FC and diffusion in WM within ARMS subjects (Wood *et al.* 2013); these studies have reported mixed results. Both increases and decreases in FC were found across multiple ICNs in ARMS subjects. In one seed-based task-free fMRI study, ARMS subjects showed increased FC within the default mode network (DMN; Shim *et al.* 2010), while another recent study found reduced FC associated with the dorsal striatum and preserved FC in the ventral striatum (Dandash *et al.* 2014). DTI studies of ARMS subjects have found reduced FA in frontal regions, the anterior limb of the internal capsule, the posterior cingulate and the angular gyrus (Hoptman *et al.* 2008; Karlsgodt *et al.* 2009; Peters *et al.* 2009; von Hohenberg *et al.* 2014). Increased FA has also been reported in the ACC and the right middle and superior frontal gyri (Hoptman *et al.* 2008) as well as in the superior longitudinal fasciculus (Schmidt *et al.* 2015). Although these inconsistent findings may reflect true heterogeneity in ARMS patients, they may also be attributed to the small sample sizes used in the aforementioned studies. Moreover, the majority of previous studies have examined ARMS patient samples in North America, Europe and Australia. There is a dearth of DTI and functional connectivity studies in East Asian populations. As culture plays an important role in defining and shaping the presentation of psychotic symptoms in patients (Laroi *et al.* 2014), here we aim to fill this gap by studying a group of Asian ARMS participants in Singapore. Another advantage of conducting this research in Singapore is the relatively low prevalence of drug use (United Nations Office on Drugs and Crime, 2008). Substance use is a problematic confound in ARMS research in Western countries (Habets *et al.* 2011; Welch *et al.* 2011) and has been shown to have the potential to affect both structural and functional brain integrity (Liao *et al.* 2012; Edward Roberts *et al.* 2014). Our recent work found no gray-matter volume reduction in substance-free ARMS subjects; this finding contrasts with results found for Western ARMS subjects with a high prevalence of drug use (Klauser *et al.* 2015). Therefore, the study of ARMS subjects who have minimal exposure to substance use allows for a more precise characterization of symptom-related brain connectivity changes.

In addition to examining the structural and functional integrity of the SN in ARMS subjects, another aim of the present study is to investigate whether any of the SN abnormalities identified in ARMS subjects could be used to predict individual clinical outcomes. Several neuroimaging studies have found baseline differences between ARMS individuals who transition to psychosis (ARMS-T) and those who do not (ARMS-NT) in cortical thickness (Tognin *et al.* 2013), WM integrity (Bloemen *et al.* 2010; Carletti *et al.* 2012) and task-related activations (Allen *et al.* 2012). These studies highlighted structural and functional changes in the prefrontal cortex, anterior insula (AI), temporal lobe and subcortical structures as potential features associated with transition. However, little is known about whether and how ICNs predict the transition to psychosis.

To this end, we tested the functional and structural SN dysconnectivity hypothesis in a relatively large East Asian sample of 96 drug-free ARMS subjects and 46 healthy controls (HCs) using both task-free fMRI and DTI approaches. We predicted that the FC and integrity of WM microstructure of SN would be reduced in ARMS individuals and that the degree of alteration would correlate with symptom severity. Within the ARMS group, we also expected that compared to ARMS-NT, the ARMS subgroup who transitioned to psychosis would have more reductions of FC and WM microstructure disruptions in the SN.

## Method

### Participants

We studied 96 ARMS subjects and 46 HCs (aged 14–29 years). The participants were assigned to the ARMS group if they met specific criteria based on the results of assessment with the Comprehensive Assessment of At-Risk Mental States (CAARMS; Yung *et al.* 2005; Yaakub *et al.* 2013). The study protocol for this investigation was approved by the National Healthcare Group Domain-Specific Review Board. Further details are included in the Supplementary Methods.

Participants were excluded from the study if they had a history of neurological or serious medical disorder, had a diagnosis of mental retardation, or were taking any antipsychotic medications. Participants with a current history of illicit substance use were also excluded from the study. Fifty-two of ARMS participants were taking prescription antidepressants. Age-matched HCs were recruited from the community if they had no history of neuropsychiatric disorder and no family history of psychosis in first-degree relatives.

### Image acquisition

The participants underwent one neuroimaging session on a 3-T Siemens Tim Trio system (Siemens, Germany). The session included the acquisition of high-resolution T1-weighted images (TR/TE = 2300/3 ms, voxel size = 1.0  ×  1.0  ×  1.0 mm^3^) during a 6-min task-free fMRI scan (TR/TE = 2000/30 ms, voxel size = 3.0  ×  3.0  ×  3.0 mm^3^; participants were instructed to close their eyes and not fall asleep) and two runs of diffusion MRI (TR/TE = 9600/107 ms, voxel size = 2.0  ×  2.0  ×  2.0 mm^3^, 30 non-collinear diffusion directions with *b* = 1000 s/mm^2^, 6 volumes at *b* = 0 s/mm^2^).

### FC analyses

The task-free fMRI data were preprocessed using procedures outlined in a previous study (Zuo *et al.* 2012) using the FMRIB Software Library (FSL; Jenkinson *et al.* 2012) and the Analysis of Functional NeuroImages software (Cox, 1996) (see Supplementary Methods). A total of 87 of the 96 ARMS participants and 37 of the 46 HCs passed both the T1 and fMRI quality control measures.

AI is the key region in the SN underlying salience processing and introceptive awareness (Seeley *et al.* 2007; Uddin, 2015). We examined SN FC integrity using the seed-based FC approach based on AI. To account for structural and functional differences in AI subregions (Mesulam & Mufson, 1982; Mutschler *et al.* 2009; Wager & Barrett, 2004) and the lateralization of dysconnectivity reported in psychosis (Wang *et al.* 2004; Kunimatsu *et al.* 2008), we estimated whole-brain voxel-wise FC using four AI seeds that covered the ventral and dorsal fields of the AI bilaterally; these seeds were chosen based on an independent meta-analysis of task-based fMRI studies of insular function (Kurth *et al.* 2010) (Supplementary Table S1, Supplementary Fig. S1). Group differences in FC to each of the four AI seeds were examined using two-sample *t* tests with a height threshold of *p* < 0.05 and cluster threshold of *p* < 0.05 family-wise error (FWE) corrected.

### Motion scrubbing

To further investigate if any observed group differences in FC were caused by head motion, we applied motion scrubbing on task-free fMRI data by computing frame displacement (FD) and variance of temporal derivative of time-courses over voxels (DVARS) from each subject's task-free fMRI data, following the steps outlined in a previous study (Power *et al.* 2012). We discarded fMRI volumes with FD > 0.2 mm, or DVARS > 0.3% (Power *et al.* 2013) and repeated the FC analysis with motion-scrubbed data.

### WM microstructural analyses

The diffusion MRI data were preprocessed and analyzed using the TBSS pipeline (Smith *et al.* 2006) following our previous approach (Cortese *et al.* 2013; Hong *et al.* 2015) (see Supplementary Methods). A total of 81 ARMS participants and 36 HCs passed the quality check for DTI data. A recent study indicated that early-stage schizophrenia was characterized by excessive extracellular-free water, which could result in a biased estimation of DTI metrics (Pasternak *et al.* 2012). We thus implemented additional steps to derive DTI metrics after accounting for partial volume effects contributed by extracellular free water. This was achieved using a modified bi-tensor model to estimate the diffusion properties of WM tissue and surrounding free water separately (Pasternak *et al.* 2009). Group differences in the skeletonized images of the free-water corrected DTI metrics (FA, AD, mean diffusivity, radial diffusivity) were examined using non-parametric permutation tests at a threshold of *p* < 0.05 (threshold-free cluster enhancement corrected) (Smith *et al.* 2006). From the thresholded *t* statistic maps, the anatomical locations of significant WM clusters were identified using the JHU WM tractography atlas (Hua *et al.* 2008).

### Correlations with clinical severity

Based on the identified regions of disrupted SN functional connectivity in ARMS subjects, we assessed the relationships between the mean FC measures in those brain ROIs and symptom severity across ARMS subjects (see  Supplementary Methods). Similarly, to evaluate the relationship between altered WM microstructure and clinical symptom severity, we extracted the subject-level mean DTI metrics from the WM clusters that showed group differences and examined their association with CAARMS scores in ARMS.

### Comparisons of FC and WM microstructure in ARMS subgroups

All ARMS subjects were followed clinically for 24 months after the neuroimaging study. During the follow-up period, 10 out of 79 ARMS subjects transitioned to psychosis (eight males; mean age = 21.5 ± 3.5 years). Psychosis transition was defined according to the criteria described in CAARMS and required the presence of frank psychotic symptoms for at least 1 week (Yung *et al.* 2005). Based on the identified ROIs showing group difference between ARMS and controls, we next compared the ROI-based FC and DTI metrics between ARMS-T and ARMS-NT subgroups using two-sample *t* tests. Age, gender, handedness and ethnicity were included as covariates in all statistical analyses.

## Results

### Participant characteristics

There were no differences in age, handedness, gender and motion parameters (both absolute displacement and frame displacement) between the ARMS and HC groups (*p* < 0.05) (Table 1, Supplementary Table S2). There was a higher proportion of ethnic Chinese individuals in the ARMS group compared to the HC group (*p* = 0.003). Furthermore, we found no differences in demographics nor motion parameters between ARMS-NT and ARMS-T groups, except a higher proportion of ethnic Indian individuals in the ARMS-NT group compared to the ARMS-T group (*p* = 0.045).

**Table 1.** Characteristics of individuals with At-Risk Mental State (ARMS) and healthy control participants in fMRI functional connectivity analysis

	ARMS (*N* = 87)		Healthy controls (*N* = 37)		Test statistics	
	*N*	%	*N*	%	χ^2^	*p*
Male	58	66.7	20	54.1	1.77	0.18
Handedness						
Right	76	87.4	32	86.5	0.99	0.61
Left	5	5.7	1	2.7		
Mixed	6	6.9	4	10.8		
Ethnicity*					14.97	0.00
Chinese	62	71.3	16	43.2		
Malay	19	21.8	9	24.3		
Indian	5	5.7	10	27.0		
Other	1	1.1	2	5.4		
Medication	52	59.8	0	0.0	–	–
	Mean	s.d.	Mean	s.d.	*t* tests	*p*
Age (years)	21.5	3.6	22.3	4.0	−0.92	0.36
Positive and Negative Syndrome Scale						
Positive subscale score	10.8	2.9				
Negative subscale score	11.9	4.0				
General psychopathology score	25.5	7.3				
Total score	48.2	12.0				
CAARMS*						
Total severity	7.3	3.4	0.2	0.4	19.38	0.00
Total frequency	8.3	4.5	0.2	0.7	16.30	0.00
Head motion parameters						
Absolute displacement (max)	0.59	0.56	0.74	0.71	−1.25	0.21
Absolute displacement (mean)	0.19	0.14	0.24	0.16	−1.77	0.08
Frame displacement (max)	0.30	0.39	0.42	0.59	−1.10	0.28
Frame displacement (mean)	0.05	0.03	0.06	0.03	−0.61	0.54

CAARMS, Comprehensive Assessment of At-Risk Mental States.

*t* tests and χ^2^ tests were used to assess group differences in continuous and discrete variables, respectively. * Represents a significant difference in ethnicity composition and CAARMS scores between ARMS subjects and healthy controls (*p* < 0.05). The demographic information of subjects included in DTI analysis is presented in  Supplementary Table S2 (81 ARMS and 29 controls overlapped with the fMRI cohort).

### Disrupted AI FC in ARMS subjects

**Fig. 1.**
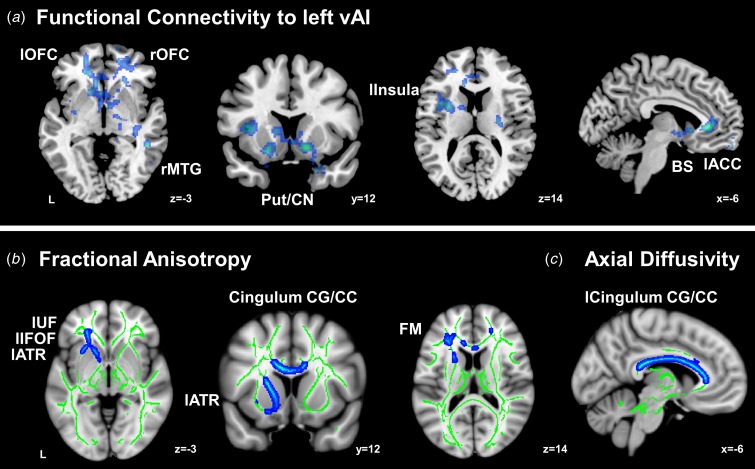
Reduced functional connectivity (FC) to the left ventral anterior insula and disrupted white-matter integrity in At-Risk Mental State (ARMS) subjects compared to healthy controls. Top row: (*a*) Group-level differences (ARMS < controls) in FC using seeds in the left ventral anterior insula were reported (at a height threshold of *p* < 0.05 and cluster threshold of *p* < 0.05 FWE corrected). No FC increase was found in ARMS compared to controls. Bottom row: white-matter tracts where ARMS subjects showed reduced fractional anisotropy (*b*) and axial diffusivity (*c*) compared to controls were reported (regions highlighted in blue) at *p* < 0.05 threshold-free cluster enhancement corrected. ACC, Anterior cingulate cortex; ATR, anterior thalamic radiation; BS, brainstem; CC, corpus callosum; CG, cingulate gyrus; CN, caudate nucleus; FM, forceps minor; IFOF, inferior fronto-occipital fasciculus; MTG, middle temporal gyrus; OFC, Orbital frontal cortex; Put, putamen; UF, uncinate fasciculus; l, left; r, right.

ARMS participants had reduced FC to the left ventral anterior insula (vAI) compared to HC subjects (Fig. 1*a*, Supplementary Table S3), involving several SN regions (left ACC, right posterior insula, bilateral OFC, bilateral putamen/caudate nucleus, right brainstem) and the right middle temporal gyrus (MTG). We found no regions of reduced FC to the other three seeds in ARMS subjects. These observations remain highly similar after motion scrubbing (Supplementary Fig. S2). No regions showing increased FC in the ARMS group with or without motion scrubbing.

### Disrupted WM microstructure in ARMS

**Fig. 2.**
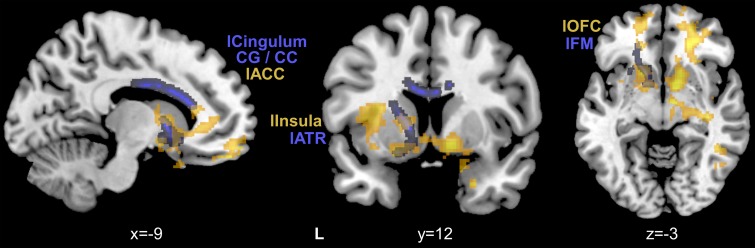
Structural dysconnectivity in At-Risk Mental State (ARMS) is closely linked to salience network functional connectivity reductions. Compared to healthy controls, ARMS participants had reduced fractional anisotropy in white-matter tracts (highlighted in blue), which were close to those brain regions (highlighted in orange) showing reduced salience network functional connectivity to the left ventral anterior insula in ARMS. ACC, anterior cingulate cortex; ATR, anterior thalamic radiation; CC, corpus callosum; CG, cingulate gyrus; FM, forceps minor; OFC, orbital frontal cortex; l, left; r, right.

We found disrupted WM microstructural measures in ARMS subjects compared to controls. Specifically, ARMS subjects had reduced FA in the left cingulum, left side of the corpus callosum, left uncinate fasciculus (UF), forceps minor, left inferior fronto-occipital fasciculus (IFOF), left superior longitudinal fasciculus, and left anterior thalamic radiation (ATR) (Fig. 1*b*, Supplementary Table S4). Similarly, reduced AD of ARMS was primarily found along the anterior to posterior axis of the bilateral cingulum/corpus callosum (Fig. 1*c*, Supplementary Table S4). WM regions that showed microstructural abnormalities in ARMS were close to those gray-matter regions that displayed reduced FC to the left vAI (Fig. 2).

Conversely, no increase in FA or AD was found in ARMS subjects compared to HCs. Additionally, we found no differences in mean diffusivity and radial diffusivity between ARMS and HC subjects.

### Correlation between WM microstructure and symptom severity

**Fig. 3.**
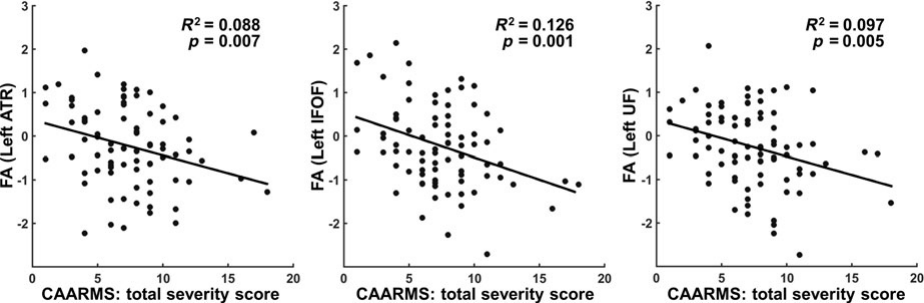
White-matter integrity disruption correlates with symptom severity. Clinical severity evaluated by Comprehensive Assessment of At-Risk Mental States (CAARMS) was negatively correlated with fractional anisotropy (FA) values in the white-matter regions with group difference (Fig. 2) (*p* < 0.05 FWE corrected). FA values in scatter plots are standardized residuals after controlling for age, gender, handedness and ethnicity. ATR, Anterior thalamic radiation; IFOF, inferior fronto-occipital fasciculus; UF, uncinated fasciculus.

Symptom severity, as measured by CAARMS total severity score, was correlated with decreased FA within the left IFOF (*r* = −0.355, *p* < 0.05 FWE corrected), the left UF (*r* = −0.311, *p* < 0.05 FWE corrected) and the left ATR (*r* = −0.297, *p* < 0.05 FWE corrected) in ARMS subjects (Fig. 3). We found no association between CAARMS total severity scores and the FC of SN regions that showed disrupted SN connectivity in ARMS.

### Difference in functional and structural disruptions between ARMS-T and ARMS-NT

**Fig. 4.**
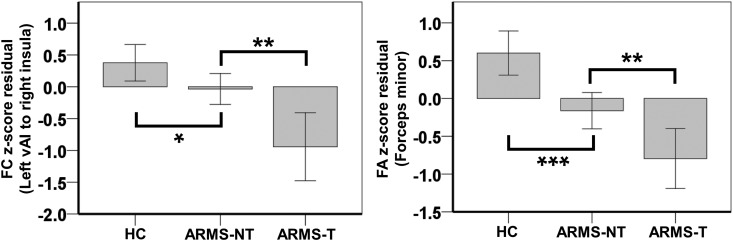
Functional and structural dysconnectivity predicted psychotic conversion in At-Risk Mental State (ARMS) subjects. Bar charts showing FC between left vAI and right insula as well as FA in the forceps minor of healthy controls (HC), ARMS subjects who transitioned to psychosis (ARMS-T) and ARMS subject who did not make the transition (ARMS-NT). FC or FA values were standardized residuals after controlling for age, gender, handedness and ethnicity. Error bars represent 95% confidence intervals. The significance of pairwise group differences is indicated by *p < 0.05, **p < 0.01 and ***p < 0.001. The comparisons between HC and ARMS-T are significant at *p* < 0.001 for both plots (not marked). All passed the multiple comparison correction (*p* < 0.05), except the contrast between HC and ARMS-NT for FC. FA, Fractional anisotropy; FC, functional connectivity; vAI, ventral anterior insula.

Among the brain regions that showed reduced FC in ARMS subjects compared to HCs, we found that FC between the left vAI and the right insula was further reduced in ARMS-T subjects compared to ARMS-NT subjects (*t* = −3.413, *p* < 0.05 FWE corrected) (Fig. 4, left). Similarly, among the WM regions that showed ARMS-associated FA reductions, the FA of the forceps minor was more reduced in ARMS-T subjects compared to ARMS-NT subjects (*t* = −2.977, *p* < 0.05 FWE corrected) (Fig. 4, right). In contrast, we found no group difference in CAARMS total severity between ARMS-T and ARMS-NT (*p* < 0.05 corrected).

### Co-morbid depression, anxiety disorder and antidepressant medication

To investigate the FC and WM microstructural differences that could be related to anxio-depressive disorders and antidepressant usage, we further compared FC and WM FA values in disrupted regions between ARMS subjects who had a concomitant diagnosis of depression and/or anxiety (*N* = 52) and ARMS subjects who did not (*N* = 35). We found no significant differences between the two groups. A similar comparison between ARMS individuals who were taking antidepressants and those who were not also did not reveal any significant differences.

## Discussion

Although there is evidence for the SN dysconnectivity hypothesis in schizophrenia, whether the hypothesis extends to at-risk subjects remains unclear. We used both task-free fMRI and DTI to test the SN dysconnectivity hypothesis in a large sample of 96 substance-free ARMS subjects. Relative to age-matched HCs, ARMS subjects showed reduced FC between the left vAI and several SN regions. The ARMS group also showed reduced FA and AD in fronto-striatal-thalamic circuits close to the regions that showed reduced FC. Furthermore, the disruptions in WM microstructure related to the severity of attenuated psychotic symptoms in ARMS subjects. These SN disruptions and the related WM microstructure alternations also predicted transition to psychosis. Taken together, our findings support the hypothesis of network-level disruptions of the salience system in ARMS participants.

### Reduced vAI FC in ARMS

In the ARMS group, key SN regions, including the right insula, left ACC, bilateral OFC, bilateral striatum, and brainstem, were found to exhibit reduced intrinsic FC with the vAI. These findings are in agreement with previous task-free fMRI studies reporting SN intrinsic connectivity disruptions in schizophrenia (Fusar-Poli, 2012; Tu *et al.* 2012; Mamah *et al.* 2013; Wood *et al.* 2013). Because the AI performs a wide range of neurocognitive functions and is functionally differentiated (Wager & Barrett, 2004; Mutschler *et al.* 2009), it is not surprising that AI subfields did not contribute equally to SN dysconnectivity in ARMS. Our findings highlighted the left vAI as the focal point of network disruptions in pre-clinical psychosis. The vAI has been shown to be involved in the regulation of emotionally related physiological states and to serve as an intermediary for functional associations between other insular regions and the limbic system (Krolak-Salmon *et al.* 2003; Mutschler *et al.* 2009). This could explain our finding that most of the FC disruptions involving the left vAI also involved regions that are closely associated with the limbic system, including the ACC, bilateral insula, and striatum regions.

Emerging evidence demonstrated dysconnectivity between SN and other ICNs in patients with schizophrenia (Jafri *et al.* 2008; Mamah *et al.* 2013; Palaniyappan *et al.* 2013; Wotruba *et al.* 2014) and reduced long-range FC in early onset schizophrenia (Yang *et al.* 2014; Jiang *et al.* 2015; Li *et al.* 2015). We found reduced FC between left vAI and right MTG, usually considered to be part of the DMN (Greicius *et al.* 2004; Fox *et al.* 2005), in the ARMS group. The impaired interactions between specific regions of SN and DMN may be reduced in ARMS participants, which suggest that persons at-risk for psychosis might have difficulty coordinating between externally guided and internally monitored states, leading to subthreshold psychotic symptoms (Menon & Uddin, 2010; Zhou & Seeley, 2014).

### Disrupted WM microstructure in fronto-striatal-thalamic circuits correlated with symptom severity in ARMS

Reduced FA along major WM tracts connecting the thalamus, basal ganglia and prefrontal cortex was observed in ARMS subjects, indicating the disruption of WM microstructure in fronto-striatal-thalamic circuits. Importantly, we found an association between WM microstructure (i.e. FA measures in the left ATR, left IFOF and left UF) and psychotic symptom severity, as measured by the CAARMS. This type of WM microstructure disruption has been reported from first-episode psychosis (Federspiel *et al.* 2006; Perez-Iglesias *et al.* 2010) to chronic illness (Liu *et al.* 2013, 2014; Oh *et al.* 2009); however, few studies have been conducted among ARMS subjects (Wood *et al.* 2013). Fronto-striatal-thalamic circuits include important neural pathways, such as the ATR and fronto-striatal circuits. The latter circuits are involved in executive and other higher cognitive domains (Van der Werf *et al.* 2003; Chudasama & Robbins, 2006). Disruptions of the ATR have been associated with cognitive abnormalities and negative symptoms in patients with schizophrenia (Mamah *et al.* 2010). One task-related PET imaging study suggested a link between dysfunction in fronto-striatal systems and prodromal signs of psychosis, especially related to executive functions (Fusar-Poli *et al.* 2010). Early fronto-striatal circuit dysfunction such as that observed here could thus contribute to cognitive impairment and reduced social functioning (Fornito *et al.* 2013; Dandash *et al.* 2014). Notably, we observed a clear trend towards greater WM disruption in the left hemisphere in ARMS. Indeed, a few studies have reported reduced FA leftward asymmetry along the anterior cingulum and UF in schizophrenia patients (Wang *et al.* 2004; Kubicki *et al.* 2007; Kunimatsu *et al.* 2008). The current findings suggest a left hemisphere-dominated WM disruption pattern in early stages of psychosis. Whether and how such pattern contributes to changes in WM cerebral asymmetry requires further validation (Oertel-Knochel & Linden, 2011).

Reduced AD was primarily observed in localized regions of the cingulum, corpus callosum and ATR. Disruptions in the cingulum were previously associated with inefficient cognitive functioning in schizophrenia (Perez-Iglesias *et al.* 2010; Tang *et al.* 2010). The cingulum is critical for efficient information processing, as it links three key brain networks (Kelly *et al.* 2008; Leech *et al.* 2011). The cingulum connects frontal regions (including SN) and posterior DMN regions (i.e. the posterior cingulate cortex), and the hippocampal portion of the cingulum projects from the posterior cingulate cortex to the limbic system (i.e. hippocampus).

The concordance between disrupted FC in the SN and WM microstructure in ARMS subjects strengthens the case for aberrant SN connectivity. Using TBSS analysis, we identified altered diffusion properties along major WM tracts, which provide critical structural support to SN function. Specifically, reduced FA and AD were evident along the left ATR, forceps minor and corpus callosum. These WM tracts project into SN regions showing reduced FC in prefrontal cortex, cingulate gyrus and striatum. Although the TBSS analysis did not directly measure the strength of structural connectivity between these regions, it evaluated the diffusion properties specifically along major WM tracts in the brain, closely approximating the integrity of structural foundation supporting FC between distant brain regions (Smith *et al.* 2006). Thus using two different neuroimaging modalities, our findings showed converging evidences supporting the SN dysconnectivity hypothesis in ARMS subjects.

### FC and WM microstructure in ARMS subjects predicted clinical progression

Based on these converging functional and structural alternations in a high-risk group, an important question is whether we could use these assays to predict individual's transition to psychosis. Here, we found specific ARMS-associated changes (i.e. FC between bilateral insula and FA of forceps minor changes at baseline) that associated with subsequent psychotic conversion. Notably, the forceps minor is a WM tract that connects the lateral and medial part of the frontal lobes bilaterally; thus could be the structural mechanism that explain the reduced functional synchrony between SN regions in the left and right hemisphere. To date, a number of studies have provided evidence that dysfunction in task-related networks (Sabb *et al.* 2010; Allen *et al.* 2012) as well as whole-brain voxel-wise WM changes (Bloemen *et al.* 2010; Carletti *et al.* 2012) may foreshadow the onset of psychosis, but few have examined the changes in ICNs or network-based WM microstructures. Our findings extended these studies by demonstrating the involvement of brain networks in task-free conditions and corresponding changes in WM tracts. Since task-free fMRI could be more readily implemented in clinical settings than task-related studies, our work demonstrates that specific neuroimaging features could potentially lead to the development of clinical biomarkers that identify ARMS subgroups with increased risk of progressing into frank psychosis (McGuire *et al.* 2015).

### Limitations and future directions

The present study had several limitations. First, although TBSS partially overcomes the misalignment of FA images in WM voxel-based analysis (Smith *et al.* 2006), WM fibers at the boundary of gray matter and WM, or deep in gray-matter regions, could be omitted from consideration. In addition, TBSS uses group-averaged WM skeleton as the proxy of major WM tracts. Future works involving high-resolution diffusion imaging and WM tractography may help to examine the direct correspondence between FC and structural connectivity as well as minimize errors due to the inter-individual differences in WM structure. Moreover, the test–retest reliability of fMRI and DTI studies should be considered when interpreting our findings. While we have demonstrated ARMS-specific functional and structural dysconnectivity, good reproducibility is the prerequisite for successfully replicating these findings and transforming them into clinical biomarkers. A multisite test–retest study reported good reproducibility of diffusion metrics derived using TBSS (Jovicich *et al.* 2014). Similarly, (Song *et al.* 2012) used seed-based correlation method and found reliable and stable FC estimations in the group of young participants (mean ICC = 0.4). However, intra- and inter-individual test–retest variability do exist in task-free fMRI analysis and might be contributed to by endogenous neural dynamics and various non-neural factors such as head motion and scanner noise (Zuo & Xing, 2014). In summary, based on a substance-free ARMS sample, we provide converging evidence, from both structural and functional connectivity analyses, to support the SN dysconnectivity hypothesis in individuals at risk for psychosis. The extent of SN disruption correlated with psychotic symptom severity and was more pronounced in subjects that developed psychosis, which can be utilized for prediction of psychosis transition.

## Supplementary material

For supplementary material accompanying this paper visit http://dx.doi.org/10.1017/S0033291716001410. click here to view supplementary material
