# Supplementary material for: Disrupted salience network functional connectivity and white-matter microstructure in persons at risk for psychosis: findings from the LYRIKS study
Source: Psychol Med. 2016 Jul 11;46(13):2771–83. doi: 10.1017/S0033291716001410 (PMC5358474; doi:10.1017/S0033291716001410)

**SUPPLEMENTARY MATERIAL**

**Supplementary Methods**

**Subject recruitment**

The ARMS participants were recruited through the Institute of Mental Health and various community mental health agencies as part of the Longitudinal Youth At-Risk Study (LYRIKS), which aims to identify individuals who are at risk for developing psychosis. Participants were assigned to the ARMS group if they met the criteria for any of the three groups described in the Comprehensive Assessment of At-Risk Mental States (CAARMS) following previous work (Yaakub *et al.*, 2013, Yung *et al.*, 2005): Group 1. Vulnerable Subgroup, which identifies individuals with a family history of psychosis or schizotypal disorder in a first-degree relative and a decline in the functioning such as a substantial drop in GAF score from premorbid level; Group 2. Attenuated Psychosis Subgroup, which identifies individuals with sub-threshold psychotic symptoms; or Group 3. Brief Limited Intermittent Psychotic Symptoms Subgroup, which identifies individuals with a recent history of frank psychotic symptoms that resolved spontaneously within 1 week. The study protocol for this investigation was approved by the National Healthcare Group Domain-Specific Review Board. Written informed consent was obtained from all participants who were at least 21 years of age and from a parent or guardian if the participant was under 21 years of age (with the participant’s consent) after a full description of the study was provided. All participants were reimbursed for their time and effort.

**Task-free functional MRI preprocessing**

The preprocessing included the following steps: 1) discarding the first 6 EPI volumes to allow for magnetic field stabilization; 2) correcting for motion; 3) time series de-spiking, spatial smoothing and grand mean scaling; 4) band-pass temporal filtering (0.01-0.1 Hz); 5) removing linear and quadratic trends; 6) co-registering the structural MRI image using Boundary-Based Registration (Greve and Fischl, 2009) and then registering the image to the Montreal Neurological Institute (MNI) 152 standard space using a nonlinear registration tool (FNIRT); and 7) regressing out 8 nuisance signals (i.e., WM, CSF signals, and 6 motion parameters). The T1 data for all subjects passed our quality control measures. A total of 87 of the 96 ARMS subjects and 37 of the 46 HC subjects passed the motion quality check of the task-free fMRI scan (i.e., maximum displacement < 4 mm).

**Diffusion-weighted image preprocessing**

The two runs of DTI scan data were concatenated. Eddy current distortion and head movement were corrected using an affine registration of diffusion-weighted images to the first b = 0 volume. Diffusion gradients were rotated to improve consistency with the motion parameters. To correct for spatial distortion due to magnetic field inhomogeneity, the first b = 0 DTI volume was nonlinearly warped with the T2-weighted image using the Automatic Registration Toolbox (Ardekani *et al.*, 2005) and then co-registered back into the native b = 0 image space. The deformation field was applied to the other DTI volumes, generating distortion-corrected DTI data. Diffusion tensors were fitted for each voxel to create subject-level FA, MD, AD, and RD images. The same 87 ARMS subjects that were included in the task-free fMRI cohort (of the 96 ARMS subjects) passed the quality check for DTI data (i.e., maximum displacement < 3 mm, and no group differences in the motion parameters). A total of 37 of the 46 HC subjects passed the quality check for DTI data. A total of 27 subjects overlapped with the 37 subjects in the task-free fMRI cohort.

**TBSS analysis**

We applied TBSS (Smith *et al.*, 2006) to conduct a voxel-wise analysis of diffusion metrics within major WM pathways. First, all of the subjects’ FA maps were registered to the FMRIB58_FA template in the MNI space image using FNIRT (Andersson *et al.*, 2007). Then, the mean FA skeleton image was created from the mean FA image; this image represented the centers of the major WM fiber tracts that were common across all subjects. The mean FA skeleton was thresholded at 0.25 to exclude non-WM regions. The aligned FA data for each subject were then projected onto the mean FA skeleton (Smith *et al.*, 2006). Similarly, the MD, AD and RD maps were nonlinearly registered to the FMRIB58_FA template and then projected onto the mean FA skeleton.

**Correlations with clinical severity**

We assessed the relationship between the FC of the left vAI and symptom severity through the following steps: 1) extracting the subject-level mean FC scores from each peak cluster in the thresholded t-stats map revealing group differences; 2) computing the standard residuals of the mean FC by removing the effects of age, gender, handedness and ethnicity; and 3) correlating the CAARMS severity scores with the FC of the left vAI across all ARMS subjects using Pearson’s correlation (p < 0.05 corrected for number of regions). The same procedure was applied to assess the relationships between SC and CAARMS scores.

**Supplementary Results**

**Effects of comorbid depression, anxiety disorder and antidepressant medications**

Within the ARMS group, individuals with anxio-depressive comorbidities (n = 67) exhibited higher CAARMS total severity scores (p = 0.013, t = 2.610) than those without (n = 20). In contrast, we found no differences in CAARMS scores between the individuals who were taking antidepressant medications (n = 52) and those who were not taking such medications (n = 35).

To examine the effects of comorbidities on FC and SC, we compared the ARMS subjects with and without a concomitant diagnosis of depression and/or anxiety in terms of all of the FC and WM integrity using the same threshold, after accounting for age, gender, handedness and ethnicity as nuisance variables. We found no group difference in the AI FC, WM integrity, and SN structural connectome nodal metrics (p < 0.05 uncorrected). Similarly, comparisons of the ARMS subjects who were taking antidepressant medications and those who were not did not reveal any differences (p < 0.05 uncorrected).

**SUPPLEMENTARY REFERENCES**

**Andersson, J. L. R., Jenkinson, M. & Smith, S. M.** (2007). Non-linear registration, aka Spatial normalisation FMRIB technical report TR07JA2. *FMRIB Analysis Group of the University of Oxford*.

**Ardekani, B. A., Guckemus, S., Bachman, A., Hoptman, M. J., Wojtaszek, M. & Nierenberg, J.** (2005). Quantitative comparison of algorithms for inter-subject registration of 3D volumetric brain MRI scans. *J Neurosci Methods* **142**, 67-76.

**Greve, D. N. & Fischl, B.** (2009). Accurate and robust brain image alignment using boundary-based registration. *NeuroImage* **48**, 63-72.

**Kurth, F., Zilles, K., Fox, P. T., Laird, A. R. & Eickhoff, S. B.** (2010). A link between the systems: functional differentiation and integration within the human insula revealed by meta-analysis. *Brain Struct Funct* **214**, 519-34.

**Smith, S. M., Jenkinson, M., Johansen-Berg, H., Rueckert, D., Nichols, T. E., Mackay, C. E., Watkins, K. E., Ciccarelli, O., Cader, M. Z. & Matthews, P. M.** (2006). Tract-based spatial statistics: voxelwise analysis of multi-subject diffusion data. *Neuroimage* **31**, 1487-1505.

**Yaakub, S. N., Dorairaj, K., Poh, J. S., Asplund, C. L., Krishnan, R., Lee, J., Keefe, R. S., Adcock, R. A., Wood, S. J. & Chee, M. W.** (2013). Preserved working memory and altered brain activation in persons at risk for psychosis. *Am J Psychiatry* **170**, 1297-307.

**Yung, A. R., Yuen, H. P., McGorry, P. D., Phillips, L. J., Kelly, D., Dell'Olio, M., Francey, S. M., Cosgrave, E. M., Killackey, E., Stanford, C., Godfrey, K. & Buckby, J.** (2005). Mapping the onset of psychosis: the Comprehensive Assessment of At-Risk Mental States. *Aust N Z J Psychiatry* **39**, 964-71.

**TABLE S1. Anterior insula seeds.**

|  | Centroid Coordinate / mm | | |  |
| --- | --- | --- | --- | --- |
| ROI | X | Y | Z | Size / cm^3 |
| Left dorsal anterior insula | -36 | 14 | 4 | 4040 |
| Right dorsal anterior insula | 36 | 20 | 0 | 3008 |
| Left ventral anterior insula | -28 | 16 | -16 | 816 |
| Right ventral anterior insula | 28 | 20 | -16 | 904 |

The clusters were presented as Figure 7 in (Kurth *et al.*, 2010) (also see Supplementary Figure S1). Coordinates are reported in MNI 152 space.

**TABLE S2.** **Characteristics of individuals with At-Risk Mental State (ARMS) and healthy control participants in DTI analysis.**

|  | **ARMS** | |  | **Healthy Controls** | |  | **Test statistics** | |
| --- | --- | --- | --- | --- | --- | --- | --- | --- |
|  | **(N = 81)** | |  | **(N = 36)** | |  |  |  |
|  | N | % |  | N | % |  | Chi-square | p |
| Male | 52 | 64.2 |  | 22 | 61.1 |  | 0.10 | 0.75 |
| Handedness |  |  |  |  |  |  |  |  |
| Right | 71 | 87.7 |  | 34 | 94.4 |  | 2.37 | 0.31 |
| Left | 5 | 6.2 |  | 0 | 0.0 |  |  |  |
| Mixed | 5 | 6.2 |  | 2 | 5.6 |  |  |  |
| Ethnicity* |  |  |  |  |  |  | 8.12 | 0.04 |
| Chinese | 59 | 72.8 |  | 21 | 58.3 |  |  |  |
| Malay | 18 | 22.2 |  | 7 | 19.4 |  |  |  |
| Indian | 3 | 3.7 |  | 6 | 16.7 |  |  |  |
| Other | 1 | 1.2 |  | 2 | 5.6 |  |  |  |
| Medication | 47 | 58.0 |  | 0 | 0.0 |  | - | - |
|  |  |  |  |  |  |  |  |  |
|  | Mean | SD |  | Mean | SD |  | t tests | p |
| Age (years) | 21.5 | 3.6 |  | 22.4 | 3.7 |  | -1.25 | 0.21 |
| Positive & Negative Syndrome Scale |  |  |  |  |  |  |  |  |
| Positive subscale score | 7.5 | 5.6 |  |  |  |  |  |  |
| Negative subscale score | 8.2 | 6.4 |  |  |  |  |  |  |
| General psychopathology score | 17.8 | 13.4 |  |  |  |  |  |  |
| Total score | 33.5 | 24.6 |  |  |  |  |  |  |
| CAARMS* |  |  |  |  |  |  |  |  |
| Total severity | 7.4 | 3.3 |  | 0.1 | 0.4 |  | 19.12 | 0.00 |
| Total frequency | 8.4 | 4.5 |  | 0.2 | 0.7 |  | 15.93 | 0.00 |

T tests and χ^2^ tests were used to assess group differences in continuous and discrete variables, respectively. * represents a significant difference in ethnicity composition and CAARMS scores between the ARMS subjects and the healthy controls (p < 0.05). Abbreviations: CAARMS = Comprehensive Assessment of At-Risk Mental States. **TABLE S3. Brain regions showing reduced functional connectivity to left ventral anterior insula (vAI) in the ARMS subjects compared to the healthy controls.**

| **Regions** | **MIN (mm)** | | | **T-score** |
| --- | --- | --- | --- | --- |
|  | **X** | **Y** | **Z** |  |
| **ARMS < Controls** |  |  |  |  |
| rMTG | 46 | -40 | 0 | 4.542 |
| lPut / CN | -20 | 12 | -14 | 3.076 |
| rOFC | 20 | 40 | -12 | 3.468 |
| rPut / CN | 10 | 6 | -10 | 3.256 |
| lACC | -6 | 36 | -2 | 3.072 |
| rInsula | 34 | -22 | 2 | 2.880 |
| **ARMS > Controls** |  |  |  |  |
| None | - | - | - | - |

Clusters representing reduced functional connectivity to left vAI in the ARMS subjects compared to the healthy controls were reported at a height threshold of p<0.05 and cluster threshold of p < 0.05 FWE corrected. Abbreviations: MTG = middle temporal gyrus, Put = putamen, CN = caudate nucleus, OFC = orbital frontal cortex, ACC = anterior cingulate gyrus, r = right, l = left.

**TABLE S4. Reduced white matter integrity in ARMS subjects.**

| **Regions** | **MIN (mm)** | | | **Analysis** | |
| --- | --- | --- | --- | --- | --- |
|  | **X** | **Y** | **Z** | **Cluster size** | **T-score** |
| Fractional anisotropy: ARMS < Controls |  |  |  |  |  |
| Left anterior thalamic radiation | -21 | 19 | 6 | 707 | 3.857 |
| Left cingulum cingulate gyrus / CC | -12 | 17 | 24 | 148 | 3.998 |
| Forceps minor | -11 | 27 | 18 | 178 | 4.329 |
| Left inferior fronto-occipital fasciculus | -27 | 33 | 9 | 358 | 3.489 |
| Left uncinate fasciculus | -19 | 19 | -9 | 154 | 3.727 |
| Axonal diffusivity: ARMS < Controls |  |  |  |  |  |
| Left anterior thalamic radiation | -15 | 1 | 6 | 112 | 5.387 |
| Right anterior thalamic radiation | -9 | -25 | 26 | 36 | 2.754 |
| Left cingulum cingulate gyrus / CC | -11 | 18 | 23 | 157 | 4.906 |
| Forceps major | 13 | -41 | 14 | 198 | 3.404 |
| Forceps minor | -11 | 27 | 18 | 628 | 4.672 |
| Fractional anisotropy: ARMS > Controls |  |  |  |  |  |
| None | - | - | - | - | - |
| Axonal diffusivity: ARMS > Controls |  |  |  |  |  |
| None | - | - | - | - | - |

White matter clusters showing reduced fractional anisotropy (FA) and axonal diffusivity (AD) in the ARMS group compared to the healthy controls, thresholded at p < 0.05 corrected (Threshold-Free Cluster Enhancement). No increased FA or AD in the ARMS subjects compared to the healthy controls was found. Abbreviations: CC = corpus callosum.

**Figure S1. Anatomical locations of anterior insular seeds.** Red = left dorsal anterior insula, green = left ventral anterior insula, blue = right dorsal anterior insula, purple = right ventral anterior insula. These seeds were defined based on a meta-analysis study (Kurth *et al.*, 2010).

**
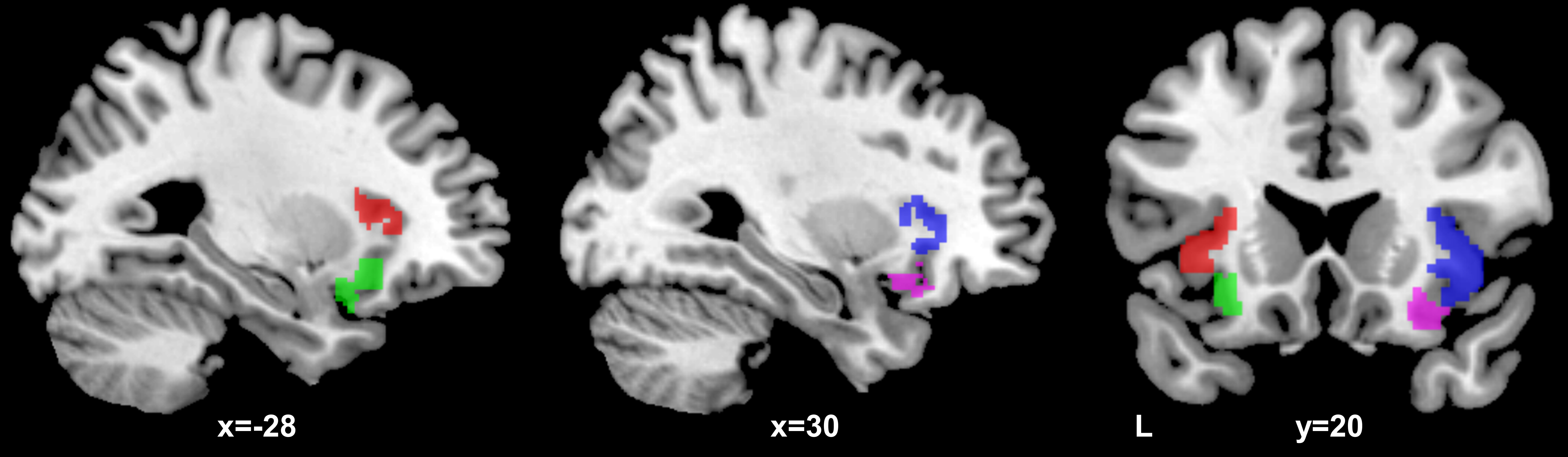
**

**Figure S2. Reduced functional connectivity to the left ventral anterior insula in ARMS subjects compared to healthy controls using motion scrubbed fMRI data**. Group-level differences (ARMS < controls) in functional connectivity (FC) using seeds in the left ventral anterior insula remained largely similar to the main results. Salience network regions (including left putamen / caudate nucleus, left anterior cingulate cortex and orbital frontal cortex) had reduced left ventral anterior insular FC in ARMS group compared to controls (height p<0.05 and cluster level p<0.05 corrected), though the rest of the regions became slightly smaller (height of p<0.05 and p<0.05 cluster-level uncorrected). No regions showing increased FC in the ARMS group after motion scrubbing. Abbreviations: OFC = orbital frontal cortex, ACC = anterior cingulate cortex, Put = putamen, CN = caudate nucleus, BS = brainstem, MTG = middle temporal gyrus, l = left, and r = right.


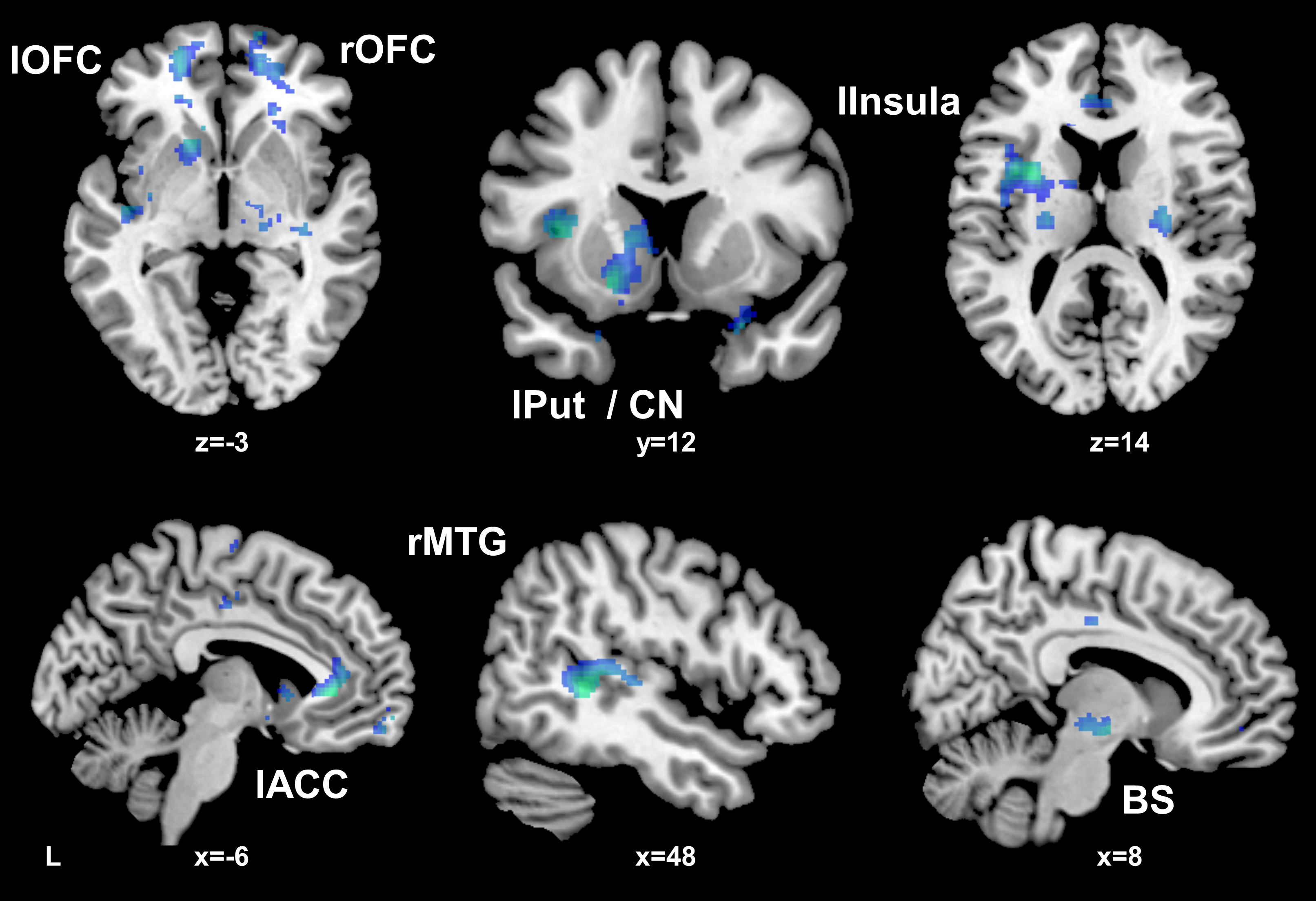

Supplement: Supplementary file 1 [file S0033291716001410sup001.docx]
